# Glucagon-Like Peptide-1 Receptor Agonists for Bile Acid Diarrhea: Emerging Evidence and Clinical Implications

**DOI:** 10.7759/cureus.105789

**Published:** 2026-03-24

**Authors:** Eunice K Omeludike, Cherechi O Nwabueze, Nneoma Ubah, Nzubechukwu Okeke, Eunice Aregbesola, Chimezie R Onyiaorah, Mesoma A Igbokwe, Meher Gujral, Ridwan A Lawal, Yetunde F Akande

**Affiliations:** 1 Internal Medicine, Piedmont Athens Regional, Athens, USA; 2 Internal Medicine, John H. Stroger, Jr. Hospital of Cook County, Chicago, USA; 3 Public Health, George Washington University, Washington, D.C., USA; 4 Medical Education, University of Nigeria, Enugu, NGA; 5 Internal Medicine, Montefiore St. Luke Hospital, Newburgh, USA; 6 Medicine, Nuvance Health, Poughkeepsie , USA; 7 Internal Medicine, University of Missouri School of Medicine, Columbia, USA; 8 Pharmacology and Therapeutics, University of Port Harcourt, Abuja, NGA; 9 Internal Medicine, Rochester General Hospital, Rochester, USA; 10 Internal Medicine, Zucker School Of Medicine at Hofstra/Northwell Health at Vassar Brothers Medical Center, Poughkeepsie, USA; 11 Internal Medicine, University of Texas Rio Grande Valley School of Medicine, Texas, USA

**Keywords:** bile acid diarrhea, bile acid malabsorption, chronic diarrhea, glp-1 receptor agonist, ibs-d, liraglutide

## Abstract

Bile acid diarrhea (BAD) is an underrecognized cause of chronic diarrhea that makes a significant contribution to the symptom burden and impaired quality of life. Despite increasing awareness, BAD is frequently misdiagnosed as diarrhoea-predominant irritable bowel syndrome (IBS-D), leading to delayed diagnosis and incomplete symptom control. Current management relies primarily on bile acid sequestrants, which are effective for many patients but limited by poor tolerability, variable adherence, and incomplete response in a subset, prompting interest in alternative therapeutic approaches targeting bile acid dysregulation.

Advances in understanding enterohepatic signaling have highlighted the role of the farnesoid X receptor-fibroblast growth factor 19 (FXR-FGF19) axis in regulating bile acid synthesis, secretion, and motility. In parallel, glucagon-like peptide-1 (GLP-1) receptor agonists, which are commonly used in the treatment of metabolic diseases, affect gastrointestinal motility, secretion, and neurohormonal signaling by mechanisms that overlap with those that are implicated in BAD. Emerging clinical studies, including randomized trials comparing GLP-1-based therapy with established bile acid sequestrants, have begun to explore their potential role in BAD, although the current evidence base remains limited and investigational.

This narrative review synthesizes peer-reviewed evidence examining the biological rationale, diagnostic context, and clinical data relevant to GLP-1 receptor agonists in BAD. Literature was identified primarily by PubMed/Medical Literature Analysis and Retrieval System Online (MEDLINE) searches supplemented by manual screening of reference lists of key reviews and clinical studies and integrated narratively because of heterogeneity of study design, exposure definitions, and outcome measures.

Current evidence suggests that GLP-1 receptor agonists represent a biologically plausible area of investigation for selected patients with persistent symptoms despite standard therapy. This review does not advocate routine clinical use but aims to contextualize emerging BAD-specific and mechanistic data to inform hypothesis generation, patient selection, and future research. Available data are still limited, and GLP-1 receptor agonists have not been established as a treatment for BAD. Further prospective studies with standardized outcomes are needed to clarify their role and inform evidence-based clinical practice.

## Introduction and background

Bile acid diarrhea (BAD) is an important and increasingly recognized cause of chronic diarrhea in adults, yet it remains underdiagnosed in routine clinical practice. Contemporary guidelines for the investigation of chronic diarrhea emphasize that bile acid-related mechanisms should be considered, particularly in patients with persistent watery diarrhea after common inflammatory, infectious, and structural causes have been excluded [1]. Despite this guidance, BAD is frequently overlooked, and many affected individuals are instead classified under symptom-based diagnoses such as functional diarrhea or diarrhea-predominant irritable bowel syndrome (IBS-D) [2,3]. This diagnostic gap contributes to prolonged symptom duration, repeated ineffective treatments, and delays in the initiation of targeted therapy.

The underrecognition of BAD is partly related to clinical overlap with IBS-D and functional diarrhea. Symptom profiles alone are insufficient to reliably distinguish bile acid-mediated diarrhea from other functional bowel disorders, as stool frequency, urgency, and loose stools are common to multiple etiologies [2,3]. Clinically, BAD may be considered primary when it arises from disordered bile acid regulation without an identifiable structural cause and secondary when it occurs in association with conditions such as ileal disease, resection, or other disorders affecting bile acid handling [3]. As a result, patients are often managed empirically without investigation of bile acid physiology, potentially missing an identifiable and treatable mechanism.

Epidemiologic data reinforce the relevance of this diagnostic challenge. A systematic review and meta-analysis demonstrated that a substantial proportion of patients meeting diagnostic criteria for IBS-D show evidence consistent with bile acid malabsorption when formally evaluated [4]. Failure to recognize this contribution may help explain why a subset of patients labeled with IBS-D respond poorly to conventional symptom-directed therapies.

BAD is also associated with meaningful symptom burden and impaired quality of life. Patient-reported outcome data indicate that individuals with BAD frequently experience urgency, increased stool frequency, fecal incontinence, abdominal discomfort, and social restriction, often persisting over long periods before effective treatment is initiated [5]. These symptoms can interfere with occupational functioning, travel, and social engagement, underscoring the broader impact of inadequately managed BAD.

Current clinical practice guidelines addressing functional diarrhea and IBS-D acknowledge mechanistic heterogeneity and recommend structured evaluation when symptoms are refractory to first-line therapy [6]. However, access to diagnostic testing and long-term therapeutic options remains variable across healthcare systems. In addition, existing treatments, while effective for many, are limited by tolerability issues, adherence challenges, and incomplete symptom control in some patients.

Against this backdrop, there is growing interest in adjunctive strategies that may address downstream physiological responses relevant to bile acid-mediated diarrhea. In this context, glucagon-like peptide-1 (GLP-1) receptor agonists have emerged as a potential area of investigation. This narrative review examines the diagnostic and biological context of BAD and synthesizes emerging evidence regarding GLP-1 receptor agonists, while maintaining a cautious framing consistent with the limited and investigational nature of the current clinical data.

While recent randomized trial data have generated interest in GLP-1 receptor agonists for BAD, the emerging literature remains fragmented across diagnostic, mechanistic, clinical, and safety domains. Existing reviews have largely addressed BAD diagnostics, enterohepatic feedback regulation via the farnesoid X receptor-fibroblast growth factor 19 (FXR-FGF19) axis, gastrointestinal actions of GLP-1 signaling, and biliary safety considerations in isolation. This conceptual separation is clinically relevant, as bile acid dysregulation primarily drives luminal and signaling abnormalities, whereas GLP-1 receptor agonists may influence downstream gastrointestinal motility and neurohormonal responses that contribute to symptom expression.

This narrative review aims to integrate these domains into a unified clinical framework to contextualize early BAD-specific trial findings, distinguish direct clinical evidence from indirect mechanistic support, and frame GLP-1 receptor agonists as downstream modulators of symptom expression rather than disease-modifying therapies targeting bile acid synthesis. By situating GLP-1-based therapies within a precision-medicine perspective, this review seeks to inform hypothesis generation, patient selection, and future BAD-specific research, with a distinction between direct clinical trial findings and indirect mechanistic or observational evidence, rather than advocate routine clinical use.

## Review

Review design

This study was conducted as a narrative literature review examining evidence on the role of GLP-1 receptor agonists in the pathophysiology and management of BAD. A narrative approach was selected to integrate heterogeneous evidence spanning physiology, translational mechanisms, diagnostic frameworks, and clinical trial data. Given the extremely limited number of BAD-specific clinical trials and the heterogeneity of available mechanistic and translational evidence, a systematic review approach would be unlikely to meaningfully increase evidentiary certainty at this stage. Accordingly, no formal quantitative synthesis, including meta-analysis or meta-regression, was performed.

Information sources

The search strategy was intentionally limited to PubMed/Medical Literature Analysis and Retrieval System Online (MEDLINE), supplemented by manual review of reference lists from authoritative narrative reviews, clinical practice guidelines, and key mechanistic or clinical studies retrieved during database searches, to prioritize clinically indexed, peer-reviewed literature most relevant to the translational and clinical focus of this narrative review.

Search strategy and timeframe

Searches were performed in PubMed/MEDLINE from database inception through January 15, 2026. Search terms focused on BAD and bile acid malabsorption, combined with terms related to GLP-1 physiology and GLP-1 receptor agonists. An example PubMed query was: (“bile acid diarrhea” OR “bile acid diarrhoea” OR “bile acid malabsorption” OR “SeHCAT” OR “FGF19” OR “C4”) AND (“GLP-1” OR “glucagon-like peptide-1” OR “GLP-1 receptor agonist” OR liraglutide OR exenatide).

Reference lists of relevant reviews and clinical studies were manually screened to identify additional pertinent literature.

Study selection approach

Titles and abstracts retrieved from PubMed/MEDLINE were screened for relevance to BAD, bile acid physiology, enterohepatic signaling, GLP-1 signaling, and clinical outcomes related to diarrhea syndromes. Full texts were reviewed when abstracts suggested direct relevance. Selection was performed by the author, with inclusion decisions based on clinical and mechanistic relevance to adult BAD, and with attention to avoiding overinterpretation when evidence was indirect.

Eligibility considerations

Eligible sources included peer-reviewed narrative reviews, systematic reviews, meta-analyses, randomized controlled trials, observational clinical studies, and selected mechanistic or translational studies with direct relevance to bile acid physiology, BAD, or GLP-1 receptor signaling in gastrointestinal contexts. Studies focused exclusively on pediatric populations or on disorders unrelated to bile acid handling were excluded unless they provided mechanistic insights applicable to adult disease. Editorials and expert reviews were included selectively when they provided clinically relevant synthesis in areas where randomized trial data remain limited.

Data synthesis and organization

Evidence was synthesized narratively and organized thematically. Sections address the clinical and diagnostic context of BAD, key bile acid signaling pathways, established therapy, and the gastrointestinal actions of GLP-1 and GLP-1 receptor agonists. Emerging BAD-specific clinical trial and translational findings were presented with explicit separation from indirect evidence drawn from related settings.

Rigor and narrative review safeguards

To reduce overinterpretation, causal language was avoided when summarizing associative, mechanistic, or hypothesis-generating evidence. Distinctions were maintained between established clinical practice, emerging therapeutic hypotheses, and investigational findings.

Ethics

This study synthesized data from published literature and did not involve human participants, animal subjects, or identifiable patient data. Ethics approval was not required.

Evidence synthesis and clinical context

Clinical Positioning of BAD Within Chronic Diarrhea Syndromes

BAD is recognized as a clinically important cause of chronic watery diarrhea in adults, and guidelines recommend considering bile acid-mediated mechanisms during structured evaluation after exclusion of common inflammatory, infectious, and structural causes [1]. Despite this guidance, BAD remains underdiagnosed, with many patients classified under functional diarrhea or IBS-D without assessment of bile acid physiology [2,3]. Symptom overlap limits diagnostic discrimination by history alone, and empiric management without targeted evaluation can delay effective therapy [2,3].

Epidemiologic data support the relevance of BAD in chronic diarrhea populations. A systematic review and meta-analysis demonstrated that a substantial proportion of patients meeting IBS-D criteria show evidence consistent with bile acid malabsorption when formally evaluated [4]. Patient-reported outcome studies highlight the burden associated with delayed diagnosis, including urgency, fecal incontinence, and social restriction [5]. Guidelines addressing functional diarrhea and IBS-D emphasize heterogeneity and recommend structured evaluation in refractory disease, within which BAD represents a key mechanistic consideration [6].

Defective Feedback Inhibition and Bile Acid Overproduction

BAD is not solely a consequence of impaired ileal reabsorption. Clinical work demonstrated that some patients exhibit defective feedback inhibition of hepatic bile acid synthesis, resulting in increased bile acid production and delivery to the colon despite an anatomically intact ileum [7]. Subsequent syntheses emphasize that dysregulated bile acid homeostasis can drive secretory diarrhea even without structural abnormalities [2].

Diagnostic evaluation of BAD

Several diagnostic tools assess different components of bile acid physiology. The ^75SeHCAT retention test quantifies bile acid retention and is one of the most studied modalities in this field [8]. Reduced SeHCAT retention identifies patients more likely to respond to bile acid-targeted therapy. In practice, SeHCAT provides a functional assessment but may be limited by availability, whereas biochemical markers such as FGF19 and C4 offer more accessible alternatives but require careful interpretation within the clinical context.

Biochemical approaches include circulating FGF19, which is associated with BAD when reduced [9], and serum 7α-hydroxy-4-cholesten-3-one (C4), a surrogate marker of hepatic bile acid synthesis that can help exclude or support BAD in chronic diarrhea and IBS-D cohorts [10]. Reviews emphasize that diagnostic strategies should be interpreted in a clinical context, considering test availability and predictive value rather than relying on symptom patterns alone [11].

Table 1 summarizes the principal diagnostic approaches used to support a diagnosis of BAD, outlining what each modality reflects physiologically, its typical clinical role, and practical considerations that may influence use in routine practice.

**Table 1:** Diagnostic approaches used to support a diagnosis of bile acid diarrhea Abbreviations: IBS-D, diarrhea-predominant irritable bowel syndrome; FGF19, fibroblast growth factor 19; C4, 7α-hydroxy-4-cholesten-3-one; SeHCAT, ^75selenium-homotaurocholic acid test. Credits: This table has been created by the authors based on the cited sources [8–10].

Diagnostic approach	What it reflects	Typical clinical role	Practical considerations	Key references
^75SeHCAT retention	Whole-body bile acid retention	Supports diagnosis and predicts response to sequestrants	Availability varies; interpretation and access can limit use	[8]
Serum FGF19	Feedback signaling related to bile acid synthesis suppression	Supports diagnosis in selected settings	Assay availability; interpretation within clinical context	[9,10]
Serum C4	Surrogate of hepatic bile acid synthesis	Supports exclusion or diagnosis in chronic diarrhea and IBS-D cohorts	Requires appropriate thresholds and clinical integration	[10]

Foundational bile acid signaling relevant to downstream mechanisms

Bile acids act as signaling molecules that regulate metabolic and gastrointestinal pathways through nuclear and membrane-bound receptors, influencing bile acid synthesis, transport, and intestinal function [12]. Experimental studies defined key molecular components of feedback inhibition, including growth factor-dependent signaling cascades involved in suppression of bile acid biosynthesis [13], the enterohepatic role of FGF15 (rodents) and FGF19 (humans) [14], and human hepatocyte evidence that bile acids activate FGF19 signaling to inhibit cholesterol 7α-hydroxylase expression [15]. These mechanisms support BAD as a disorder of disrupted enterohepatic signaling and provide context for therapeutic strategies beyond intraluminal bile acid binding.

Colonic bile acid exposure and symptom generation

Excess delivery of bile acids to the colon contributes to diarrhea in BAD. Physiological reviews describe bile acids as active signaling molecules that influence secretion, motility, and epithelial transport [16]. Clinical studies show increased colonic bile acid exposure in subsets of IBS-D patients and associations between bile acid delivery and symptoms [17]. Neurohumoral pathways also contribute, including bile acid activation of receptors such as TGR5, influencing enteric neural activity and motility patterns that may contribute to symptom generation in bile acid diarrhea [18].

Diagnostic tools in clinical practice: strengths and constraints

Although mechanistic understanding has advanced, implementation in routine practice remains uneven. Reviews describe SeHCAT utility but note practical constraints and variation in availability [19]. Prospective comparisons suggest serum-based measures can support diagnosis where nuclear medicine testing is unavailable [20]. Expert syntheses emphasize that no single modality fully captures BAD heterogeneity and that results should be interpreted alongside clinical features and treatment response [21]. Recent systematic reviews highlight progress and persistent gaps in access and standardization [22], and editorial commentary emphasizes pragmatic diagnostic approaches for routine care [23].

Established therapeutic approaches: bile acid sequestration

Given that excess colonic bile acid exposure is a key driver of symptoms in BAD, treatment strategies have focused on reducing intraluminal bile acid levels. Bile acid sequestrants remain the cornerstone of BAD management [24]. A meta-analysis of randomized trials supports improvement in stool frequency and consistency in many patients, with variable response rates and heterogeneity across studies [25]. Controlled physiological studies demonstrate that sequestrants can modify intestinal transit and bowel function [26]. Randomized trials in Crohn’s disease associated with bile acid malabsorption support the benefit of colesevelam compared with placebo [27]. Phase 4 randomized trial data extend evidence to idiopathic BAD, supporting efficacy and short-term safety [28]. However, gastrointestinal tolerability issues, including bloating and constipation, along with pill burden and adherence challenges, contribute to discontinuation and incomplete symptom control in some patients [29,30].

Table 2 provides an overview of established treatment approaches for bile acid diarrhea discussed in this review, summarizing their primary mechanistic targets, the nature of the supporting evidence, and commonly described limitations in clinical practice.

**Table 2:** Established treatments for BAD and commonly described limitations Abbreviations: BAD, bile acid diarrhea; RCTs, randomized controlled trials Credits: This table has been created by the authors based on the cited sources [24–30].

Treatment approach	Mechanistic target	Evidence base summarized in this review	Commonly described limitations	Key references
Bile acid sequestrants (class)	Reduces colonic bile acid exposure via intraluminal binding	Supported by guideline recommendations and trial/meta-analytic evidence	Tolerability, pill burden, adherence, and incomplete response in some patients	[24,25,29,30]
Colesevelam (specific agent)	Bile acid binding with effects on bowel function and transit	Physiologic studies and RCTs including phase 4 data in idiopathic BAD	Similar real-world tolerability and adherence constraints; not upstream disease modifying	[26–28]

Therapeutic limitations and unmet clinical needs

Sequestrants do not directly address upstream dysregulation of bile acid synthesis or broader enteroendocrine signaling. Some patients remain symptomatic despite treatment, and although bile acid sequestrants are commonly recommended as first-line therapy in clinical guidance, expert reviews highlight ongoing contention regarding selection, long-term adherence, and approaches for nonresponders or those who do not tolerate therapy, with variability in implementation across clinical settings [30]. These limitations support continued investigation of adjunctive strategies that may modulate downstream motility, secretion, or neurohormonal responses relevant to BAD pathophysiology.

Physiological actions of GLP-1 in the gastrointestinal tract

GLP-1 is an incretin hormone secreted by enteroendocrine L-cells of the distal small intestine and colon in response to nutrient exposure. Beyond metabolic effects, GLP-1 influences gastrointestinal motility, secretion, and neural signaling [31]. In this context, modulation of gastrointestinal motility represents a potential mechanism by which GLP-1 signaling may counter bile acid-induced diarrheal symptoms. These actions are mediated through peripheral GLP-1 receptors on enteric neurons and smooth muscle and through central pathways involving vagal afferents. Mechanistic reviews describe GLP-1 as a regulator of gastric emptying and small intestinal transit with inhibitory effects on coordinated motor activity [32]. Human physiological studies demonstrate suppression of gastrointestinal motility and inhibition of migrating motor complex activity in healthy subjects and patients with irritable bowel syndrome [33]. Pharmacologic syntheses describe region-specific effects throughout the gut, including delayed proximal transit and altered distal motor patterns [34].

Intersection between GLP-1 signaling, bile acids, and enteroendocrine regulation

Bile acids and GLP-1 signaling intersect at multiple levels within the enterohepatic axis. Reviews of gastrointestinal actions of GLP-1-based therapies describe interactions among bile acids, enteroendocrine signaling, and motility regulation [35]. Neurohormonal models of IBS emphasize that altered enteroendocrine signaling contributes to dysregulated motility and visceral sensation [36]. These pathways suggest that GLP-1 receptor agonists could influence BAD by modifying downstream physiological responses to bile acid exposure rather than by directly reducing bile acid synthesis. This framing supports consideration of GLP-1-based therapy, if studied further, as a mechanistically distinct adjunct rather than a replacement for bile acid binding therapy.

Current Evidence Map (GLP-1 Receptor Agonists in BAD)

Established BAD care: BAD is evaluated using clinical context and available diagnostic testing, and bile acid sequestrants remain first-line therapy in current clinical guidelines. While effective for many patients, these agents are limited in practice by tolerability, adherence challenges, and incomplete symptom control in a subset of individuals [24,29,30].

Direct BAD-specific evidence: Direct clinical evidence supporting GLP-1 receptor agonists in BAD currently centers on a single randomized, double-blind, active-comparator trial evaluating liraglutide versus colesevelam, with accompanying translational analyses examining bile acid profiles in treated participants [37]. These data provide an initial clinical signal based on short-term, symptom-based outcomes but remain investigational and insufficient to define routine clinical use or long-term therapeutic strategy.

Indirect evidence: Additional support derives from mechanistic studies describing gastrointestinal actions of GLP-1 and from related clinical contexts involving altered intestinal transit or bile acid handling, such as short bowel syndrome or post-ileal resection states. While these findings support biological plausibility, they are indirect and should not be considered equivalent to BAD-specific clinical evidence [31].

Emerging Clinical Evidence for GLP-1 Receptor Agonists in BAD

Clinical interest in GLP-1 receptor agonists for BAD was catalyzed by a randomized, double-blind, active-comparator trial evaluating liraglutide versus colesevelam. This non-inferiority study reported symptomatic improvement with liraglutide comparable to colesevelam over the short treatment period using symptom-based outcomes and provided the first direct clinical signal of potential relevance in BAD [37]. The trial duration and endpoint selection do not permit conclusions regarding long-term efficacy, durability of response, or disease-modifying effects. Expert commentary emphasized that GLP-1 receptor agonists should be viewed as investigational and mechanistically informative rather than immediate replacements for established therapy [38].

Translational work in trial participants reported that liraglutide and colesevelam changed serum and fecal bile acid profiles, consistent with mechanistic differences between GLP-1 receptor activation and intraluminal bile acid binding [39]. Trial protocols provide methodological context for ongoing and planned studies designed to further isolate these effects under controlled conditions [40].

Extension to related gastrointestinal conditions: Indirect support for GLP-1 receptor agonist effects on diarrheal states comes from related contexts. Reviews of BAD diagnostics and management note interest in therapies that influence motility and enteroendocrine signaling in refractory disease [41]. Clinical studies of exenatide in short bowel syndrome reported improvements in stool output and intestinal function [42]. Literature reviews and case-based syntheses describe emerging uses of GLP-1 receptor agonists following ileal resection [43]. Trials of GLP-1 analogues such as glepaglutide in short bowel syndrome demonstrate modulation of the bile acid-FXR-FGF19 axis, reinforcing links between GLP-1-based therapies and enterohepatic regulation [44]. Editorial commentary frames these observations within broader discussions of disease-modifying therapy in intestinal failure, while remaining separate from BAD-specific evidence [45].

Safety considerations and biliary effects: Safety considerations are central when evaluating therapies that influence gastrointestinal motility and bile-related physiology. Large observational analyses have reported an association between GLP-1 receptor agonist use and increased risk of gallbladder and biliary diseases in metabolic populations [46], with population-based studies suggesting variability across subgroups and body mass index categories [47]. Mechanistic reviews have proposed potential links between GLP-1 receptor activation, gallbladder hypomotility, and bile stasis, while emphasizing that causality remains uncertain [48].

An important additional consideration is the potential confounding effect of weight loss itself. Rapid or substantial weight reduction is an established risk factor for gallstone formation and may contribute to biliary events observed in metabolic populations treated with GLP-1 receptor agonists. Most available safety data derive from individuals with obesity or type 2 diabetes, in whom medication-associated weight loss is common. Whether similar biliary risk profiles apply to patients with bile acid diarrhea, who are often not obese and may experience different patterns of weight change, remains uncertain, supporting cautious extrapolation of these findings to BAD populations.

Implications for precision medicine in BAD: Recent syntheses describe BAD as a disorder suited to precision medicine approaches, given identifiable mechanisms and variable treatment response [49]. Contemporary appraisals emphasize integrating biomarkers, symptom profiles, and mechanistically targeted therapies to improve outcomes in chronic diarrhea [50]. Within this framework, GLP-1 receptor agonists represent an emerging, hypothesis-driven therapeutic option that warrants further BAD-specific study before routine clinical integration.

Discussion

This narrative review integrates key aspects of BAD relevant to chronic watery diarrhea and examines emerging evidence that GLP-1 receptor agonists may have clinical relevance in selected patients. Contemporary guidance for chronic diarrhea evaluation emphasizes consideration of bile acid-related mechanisms once common inflammatory, infectious, and structural causes have been excluded [1]. However, BAD often remains embedded within symptom-based diagnostic categories such as IBS-D or functional diarrhea, where symptom overlap can obscure identification of the underlying mechanism [2,3,6].

Evidence supporting the clinical importance of BAD includes meta-analytic data demonstrating that a meaningful proportion of patients meeting IBS-D criteria exhibit findings consistent with bile acid malabsorption on formal testing [4]. Patient-reported outcome data further indicate that delayed recognition is associated with persistent urgency, incontinence, and social restriction [5].

From a mechanistic perspective, BAD is increasingly understood as a disorder of dysregulated enterohepatic feedback rather than solely impaired ileal reabsorption. Studies describing defective feedback inhibition support the concept that some patients exhibit increased bile acid synthesis and delivery to the colon despite the absence of overt ileal disease [7,12]. Foundational signaling work has elucidated the pathway by which intestinal bile acid sensing regulates hepatic bile acid synthesis via FGF15/FGF19 signaling and downstream suppression of CYP7A1 [13,14,15].

In routine clinical practice, diagnostic evaluation of BAD remains constrained by variable access and implementation challenges. Although established diagnostic approaches are well supported by evidence, practical limitations related to availability, cost, and integration into standard care pathways continue to restrict their use in many settings [19,20]. Expert syntheses emphasize the need for pragmatic, context-appropriate diagnostic strategies that balance mechanistic accuracy with feasibility in routine practice [21-23].

Therapeutically, bile acid sequestrants remain first-line therapy [24]. Evidence includes randomized trials, mechanistic studies, and phase 4 data supporting efficacy and short-term safety [25-28]. However, tolerability, adherence challenges, and incomplete symptom control are consistently described in real-world practice [29,30].

Within this context, GLP-1 receptor agonists are of interest because GLP-1 has gastrointestinal actions that may counter mechanisms contributing to diarrhea. Mechanistic and physiological evidence describes modulation of motility and neurohormonal signaling [31-33], and pharmacologic syntheses outline region-specific actions throughout the gut and bidirectional interactions relevant to enteroendocrine regulation [34,35]. Direct BAD-specific clinical evidence remains limited but includes randomized trial data showing symptomatic improvement during liraglutide treatment compared with colesevelam over a short treatment period using symptom-based endpoints, with accompanying commentary appropriately framing the findings as investigational [37,38]. Translational studies suggest mechanistic differences between GLP-1 receptor agonists and bile acid sequestrants, supporting biologically plausible but distinct pathways of action [39,40].

Supportive evidence from related gastrointestinal contexts, including short bowel syndrome and post-ileal resection states, should be interpreted as indirect [41-45]. Safety considerations remain important. Observational studies report associations between GLP-1 receptor agonist use and biliary disease [46,47], with mechanistic syntheses emphasizing uncertainty regarding causality [48]. Most safety data derive from metabolic populations, and extrapolation to BAD populations without these characteristics should remain cautious.

Overall, GLP-1 receptor agonists represent a biologically plausible, hypothesis-driven adjunctive strategy for selected patients with BAD who remain symptomatic despite optimized standard therapy. Defining durability of response, patient selection, and long-term safety will be essential before broader clinical integration.

Limitations

This review is narrative in design and does not provide exhaustive literature capture, formal risk-of-bias assessment, or quantitative synthesis. Clinical evidence evaluating GLP-1 receptor agonists specifically in BAD remains limited, with available trials characterized by modest sample sizes, short follow-up, and selected populations. Some mechanistic and supportive clinical insights are drawn from related gastrointestinal contexts rather than BAD-specific cohorts, requiring cautious interpretation. Heterogeneity in diagnostic approaches, outcome measures, and treatment response definitions further limits cross-study comparability. Accordingly, conclusions should be interpreted as exploratory and hypothesis-supporting rather than practice-defining.

## Conclusions

BAD is a common, underrecognized, and mechanistically defined contributor to chronic watery diarrhea that is frequently misclassified within functional bowel disorders. While bile acid sequestrants remain standard first-line therapy, real-world limitations related to tolerability, adherence, and incomplete symptom control highlight an unmet need in a subset of patients. Advances in understanding enterohepatic feedback and gut hormone signaling have prompted interest in GLP-1 receptor agonists as a potential adjunctive or alternative approach. Early clinical and translational evidence suggests biological plausibility and preliminary symptomatic benefit, but the current evidence base remains limited. At present, GLP-1 receptor agonists should be viewed as investigational in BAD, warranting further study rather than routine clinical adoption. Future research should focus on BAD-specific trials with standardized diagnostics, longer follow-up, and clearly defined patient selection to clarify the therapeutic role.
